# Association between a clinical pharmacist–led multimodal remote medication intervention and individualized treatment outcomes in patients with type 2 diabetes mellitus

**DOI:** 10.3389/fendo.2026.1867163

**Published:** 2026-08-04

**Authors:** Yuanfen Zhou, Xin Liu, Kaifeng Tang, Yiyun Cai, Qimei Yang, Bangfu Wang

**Affiliations:** 1 Department of Pharmacy, The First People’s Hospital of Zunyi, The Third Affiliated Hospital of Zunyi Medical University, Zunyi, China; 2 Adverse Drug and Medical Device Reaction Monitoring Center, Zunyi Administration for Market Regulation, Zunyi, China; 3 Department of Pharmacy, Zunyi Infectious Disease Hospital, Zunyi, China

**Keywords:** clinical pharmacist, individualized therapy, medication adherence, multimodal, remote medication intervention, type 2 diabetes mellitus

## Abstract

**Objective:**

To evaluate the association between a clinical pharmacist-led multimodal remote medication intervention and glycemic control, insulin resistance and estimated β-cell function, medication adherence, and individualized treatment outcomes among patients with type 2 diabetes mellitus (T2DM).

**Methods:**

A single-center, retrospective, controlled study design was used. A total of 163 patients with T2DM treated in the Endocrinology Department of our hospital between January 2022 and December 2024 were included and allocated, according to the type of pharmaceutical service actually received, to an intervention group (IG, n=85; electronic medical records confirmed receipt of a multimodal remote medication intervention) or a control group (CG, n=78; standard pharmaceutical care). Glycemic indices (HbA1c, FPG, 2hPG), lipid profile and blood pressure, HOMA-IR, HOMA-β, MMAS-8 score, individualized target-attainment rates, and adverse drug reaction data were extracted from the electronic medical record system at baseline and 6 months after intervention, and subgroup analyses were performed according to age, insulin use, and baseline medication adherence. Analysis of covariance (ANCOVA), 1:1 nearest-neighbor propensity score matching, inverse probability of treatment weighting, and Bonferroni correction were used to evaluate the robustness of the findings; subgroup analyses were exploratory.

**Results:**

Baseline characteristics were comparable between the two groups (all P>0.05). After intervention, HbA1c, FPG, and 2hPG were all significantly lower in the IG than in the CG (all P<0.001; ANCOVA-adjusted between-group differences: HbA1c −0.72%, FPG −0.96 mmol/L, 2hPG −1.59 mmol/L). TC, TG, LDL-C, SBP, and DBP were all significantly lower, and HDL-C significantly higher, in the IG (all P<0.05). HOMA-IR was significantly lower and HOMA-β significantly higher in the IG (both P<0.001). MMAS-8 scores and the proportion of patients with high adherence were significantly greater in the IG (P<0.001). HbA1c attainment, LDL-C attainment, and composite triple-target attainment rates were all higher in the IG (all P<0.01). The overall incidence of adverse drug reaction events was lower in the IG (P<0.05). Exploratory subgroup analyses showed that, within strata defined by age, insulin use, and baseline adherence, the direction of between-group differences in the primary outcomes was generally consistent. Results of the PSM cohort (n=59 pairs) and the IPTW sensitivity analysis were directionally consistent with the primary analysis.

**Conclusion:**

In this single-center retrospective study, a clinical pharmacist-led multimodal remote medication intervention was associated with more favorable glycemic and cardiometabolic indices, lower HOMA-IR, higher estimated HOMA-β, higher medication adherence, and higher attainment of individualized treatment targets. Because of the non-randomized, observational design, and because HOMA indices are indirect surrogate markers, these associations do not establish causality, the independent contribution of any single intervention component, or recovery of β-cell function.

## 1 Introduction

Type 2 diabetes mellitus (T2DM) is a chronic metabolic disorder characterized by persistent hyperglycemia, with a globally rising prevalence over recent decades (1, 2). Beyond its profound impact on quality of life, T2DM drives a spectrum of serious complications—including cardiovascular disease, chronic kidney disease, retinopathy, and neuropathy—imposing substantial burdens on patients, families, and healthcare systems (3, 4). Pharmacological management of T2DM typically involves complex multidrug regimens comprising oral hypoglycemic agents, insulin, lipid-lowering drugs, antihypertensives, and antiplatelet therapy, all of which demand a high degree of patient self-management (5). Poor medication adherence is prevalent in this population; approximately half of patients with chronic diseases fail to take medications as prescribed, significantly compromising glycemic attainment and prognosis (6, 7). Furthermore, marked interindividual variability in T2DM—driven by age, comorbidities, and other patient-specific factors—renders uniform treatment approaches increasingly inadequate, making individualized management the cornerstone of contemporary diabetes care (8).

Clinical pharmacists, as integral members of multidisciplinary care teams, play an increasingly important role in the medication management of chronic disease. Systematic reviews and meta-analyses have demonstrated that pharmacist-led interventions can effectively improve blood pressure, lipid profiles, and medication adherence (9). However, traditional face-to-face pharmaceutical care is constrained by time, geography, and resource availability, limiting the continuity and frequency of medication management that patients require (10). The rapid growth of mobile internet, smart devices, and telemedicine technology has opened new avenues for delivering continuous, patient-centered pharmaceutical care beyond spatial and temporal boundaries (11). Multiple studies have shown that pharmacist-led remote interventions can meaningfully improve glycemic control and medication-taking behavior in patients with T2DM (12). Multimodal remote medication intervention—integrating smartphone applications, video consultations, and other digital modalities into a closed-loop management system encompassing prescription review, medication counseling, efficacy monitoring, adverse-reaction surveillance, and lifestyle guidance—may, through multichannel information integration and individualized feedback, achieve greater benefit than a single-modality intervention (13). Nevertheless, systematic evidence on the association between such interventions and individualized T2DM outcomes remains limited, particularly with respect to mechanistic evaluation of insulin resistance and estimated β-cell function and to differential benefit across patient subgroups.

Against this background, this retrospective study compared a multimodal remote medication intervention with standard pharmaceutical care with respect to glycemic control, metabolic indices, medication adherence, individualized target attainment, and adverse drug reaction events in patients with T2DM, and performed exploratory subgroup analyses stratified by age, insulin use, and baseline adherence. This study was designed to describe the association between the pharmaceutical care model and these outcomes, rather than to establish causality, demonstrate a biological mechanism, or isolate the effect of any single intervention component.

## 2 Methods

### 2.1 Study design

This was a single-center retrospective controlled study approved by the Ethics Committee of Zunyi First People’s Hospital (Approval No. 2025LL18). Given the retrospective nature of the study, the requirement for written informed consent was waived. The study was conducted in accordance with the Declaration of Helsinki. Using the Hospital Information System (HIS) and the Prescription Automatic Screening System (PASS), patients with T2DM treated in the Endocrinology Department between January 2022 and December 2024 were identified; 196 patients entered secondary screening.

#### 2.1.1 Inclusion criteria

(1) Age ≥18 years, meeting the diagnostic criteria for T2DM as defined by the Chinese Guidelines for the Prevention and Treatment of Type 2 Diabetes Mellitus (2020 edition) (14); (2) diabetes duration ≥1 year with current regular use of oral hypoglycemic agents or insulin; (3) proficient use of a smartphone with basic literacy; (4) regular follow-up in the Endocrinology Department for at least 6 months with complete electronic medical records.

#### 2.1.2 Exclusion criteria

(1) Type 1 diabetes mellitus, gestational diabetes, or other specific diabetes subtypes; (2) severe cardiac, hepatic, or renal insufficiency, or active malignancy; (3) severe cognitive impairment, psychiatric disorder, or inability to perform self-care; (4) systemic corticosteroid use within the preceding 3 months; (5) loss to follow-up or missing primary outcome data during the observation period; (6) acute diabetic complications at enrollment (e.g., diabetic ketoacidosis, hyperosmolar hyperglycemic state).

**Figure 1:**
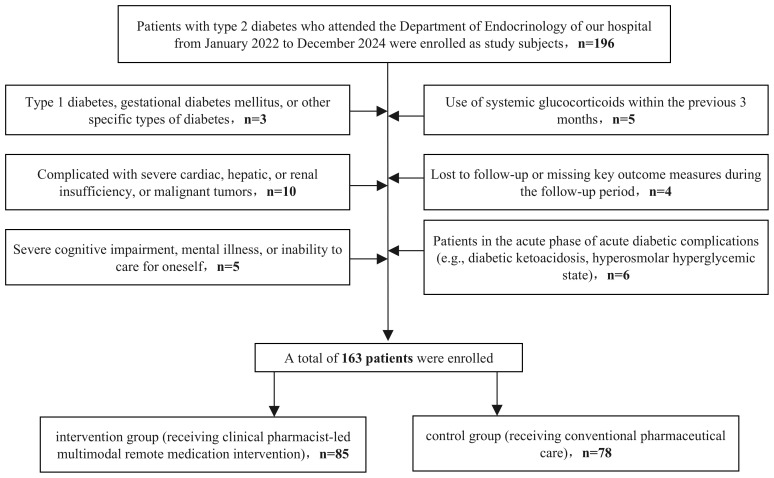
Study selection flowchart.

After applying these criteria, 163 patients were enrolled and allocated to the intervention group (clinical pharmacist-led multimodal remote medication intervention, n=85) or the control group (standard pharmaceutical care, n=78) according to the type of pharmaceutical service received as documented in medical records. The study flow is presented in **Figure 1**.

### 2.2 Interventions

#### 2.2.1 Control group

The control group received standard pharmaceutical care. According to HIS records, CG patients received verbal medication counseling from dispensing pharmacists at the outpatient pharmacy window at the time of prescription pickup, covering drug names, doses, administration schedules, and common adverse effects. Patients could obtain general pharmaceutical advice via outpatient revisits or telephone. No structured proactive follow-up, remote medication reminders, or continuous individualized regimen assessment was documented in the medical records of CG patients. Therefore, the two groups differed not only in the delivery channel of pharmaceutical service, but also in the intensity, degree of structuring, and continuity of pharmacist contact.

#### 2.2.2 Intervention group

Based on review of HIS records, pharmacist work logs, and electronic medical records, all IG patients had documented receipt of a systematic, multimodal remote pharmaceutical service delivered by pharmacists with at least 3 years of experience in diabetes-specialty pharmacy practice, all of whom completed a standardized 16-hour training program before the start of the program; training covered current guidelines and pharmacotherapy, remote communication skills, management of hypoglycemia, dose adjustment for hepatic and renal function, drug–drug interactions, data security, and standardized documentation. The service comprised four components: (1) *Mobile health management platform*: IG patients uploaded blood glucose data, submitted medication records, and communicated with pharmacists through a hospital-linked mobile health application; all data were synchronized to the pharmacist workstation. (2) *Video follow-up*: Clinical pharmacists conducted structured video consultations with patients to assess medication efficacy, analyze glycemic fluctuations, identify adverse reactions, and provide lifestyle guidance; visit summaries were archived in the HIS. The first video follow-up was scheduled within 7 days of enrollment and lasted approximately 20–30 minutes; thereafter, a structured video consultation was conducted approximately every 4 weeks, for a total of 6 sessions over 6 months, each lasting 15–20 minutes. (3) *Real-time medication reminders and alert management*: The mobile platform delivered a medication reminder 10 minutes before each scheduled dose according to the individual prescription, with a second reminder issued if the dose was not confirmed within 30 minutes; a weekly reminder was sent every Sunday at 20:00 prompting patients to upload glucose and medication-taking records. In response to abnormal glucose readings (e.g., hypoglycemia, persistent hyperglycemia) or declining adherence, pharmacists delivered targeted reminders and interventions to patients via instant messaging, with all communications archived. (4) *Individualized medication review*: Following each outpatient visit or remote follow-up, pharmacists documented a structured review of each patient’s regimen in the HIS, integrating current HbA1c, glucose profile, hepatic and renal function, lipid, and blood pressure data to generate individualized recommendations—including dose optimization, changes in administration timing, or drug substitution (14, 15)—which were reviewed and approved by the attending physician before implementation. Among the 85 IG patients, a total of 326 individualized medication-adjustment recommendations were documented, of which 300 (92.02%) were adopted by attending physicians; the 26 recommendations that were not adopted were primarily attributable to patient preference (n=11), financial considerations (n=8), or contraindications (n=7).

The observation period was 6 months for both groups, after which all outcome measures were reassessed.

### 2.3 Outcome measures

#### 2.3.1 Baseline characteristics

Age, sex, BMI, diabetes duration, education level, comorbidities (hypertension, dyslipidemia, coronary heart disease), smoking history, insulin use, and number of concomitant medications were extracted from the HIS.

#### 2.3.2 Primary outcomes (12 indices in total, included in the Bonferroni multiple-testing correction)

(1) Glycemic indices: HbA1c, fasting plasma glucose (FPG), and 2-hour postprandial glucose (2hPG). HbA1c was measured by high-performance liquid chromatography; FPG and 2hPG were measured by the hexokinase method. (2) Lipid profile: total cholesterol (TC), triglycerides (TG), low-density lipoprotein cholesterol (LDL-C), and high-density lipoprotein cholesterol (HDL-C). (3) Blood pressure: systolic blood pressure (SBP) and diastolic blood pressure (DBP). (4) Insulin resistance and β-cell function: Fasting insulin (FINS) was extracted from the laboratory system. The insulin resistance index was calculated as HOMA-IR = (FINS × FPG)/22.5, and the β-cell function index as HOMA-β = [20 × FINS/(FPG − 3.5)] × 100% (16). HOMA-IR and HOMA-β are both indirect surrogate indices derived from fasting measurements and are not equivalent to directly measured insulin sensitivity, β-cell function, or recovery of β-cell function. (5) Adherence: The Morisky Medication Adherence Scale–8 items (MMAS-8) score (17) was extracted from outpatient medical records, as MMAS-8 assessment is a routine component of follow-up documentation in our Endocrinology Department. Total scores range from 0 to 8, with >7 indicating high adherence, 6–7 moderate adherence, and <6 low adherence.

#### 2.3.3 Secondary outcomes

Individualized treatment target attainment: Per the ADA 2024 Standards of Care and the Chinese Guidelines for the Prevention and Treatment of Type 2 Diabetes Mellitus (14, 15), HbA1c attainment was defined as <7.0%; blood pressure attainment as SBP <130 mmHg and DBP <80 mmHg; LDL-C attainment as <1.8 mmol/L for patients with established atherosclerotic cardiovascular disease (ASCVD) or high cardiovascular risk, and <2.6 mmol/L for others. Composite triple-target attainment required simultaneous achievement of all three targets.

Drug-related adverse events: Hypoglycemia (blood glucose 3.0–3.9 mmol/L, per American Diabetes Association 2023 criteria) and severe hypoglycemia (<3.0 mmol/L), gastrointestinal adverse reactions, skin reactions, and hypotension (per the 2020 Chinese Guidelines for the Prevention and Treatment of Hypertension and the T2DM prevention guidelines, with a general target of <130/80 mmHg; for patients over 80 years of age or those at risk of orthostatic hypotension, the target could be individualized to <140/90 mmHg) were extracted from medical records, the adverse drug reaction reporting system, and emergency department and inpatient records.

#### 2.3.4 Quantification of intervention engagement

Participation level was defined according to the post-intervention MMAS-8 score: high participation (MMAS-8 >7), moderate participation (6–7), and low participation (<6). Effective participation was defined as an improvement in MMAS-8 of ≥1 point from baseline.

#### 2.3.5 Subgroup analyses

Three exploratory subgroup analyses were performed: (1) age stratification (<65 years vs. ≥65 years); (2) insulin use (users vs. non-users); (3) baseline medication adherence (MMAS-8 <6 [low adherence] vs. MMAS-8 ≥6 [moderate-to-high adherence]). Within each subgroup, the primary outcomes were compared between the IG and the CG. Because no formal interaction tests were performed, within-subgroup P values are used only to describe the direction of the association within each subgroup and should not be used to compare the strength of the association across subgroups or to identify a subgroup that derives greater benefit.

### 2.4 Statistical analysis

Data were analyzed using SPSS version 26.0. (1) Descriptive statistics: Continuous variables conforming to a normal distribution (assessed by the Shapiro–Wilk test) are expressed as mean ± standard deviation and were compared using independent-samples t-tests; categorical variables are expressed as frequencies and percentages [n(%)] and were compared using the chi-square (χ²) test, with Fisher’s exact test applied when any expected cell count was <5. (2) Propensity score matching: 1:1 nearest-neighbor matching was performed with a caliper width of 0.2 standard deviations of the logit propensity score (caliper=0.0892), matching on age, sex, BMI, diabetes duration, and other covariates, yielding 59 matched pairs (n=118); all covariate standardized mean differences (SMDs) were <0.1 after matching, indicating good matching quality. (3) Inverse probability of treatment weighting: Stabilized inverse probability weights were used, with extreme weights truncated at the 99th percentile to improve stability. (4) Analysis of covariance: For continuous primary outcomes, ANCOVA models were constructed using the corresponding baseline value of each outcome as a covariate, together with age and diabetes duration for multivariable adjustment; adjusted between-group differences and 95% confidence intervals are reported. (5) Multiple testing: Bonferroni correction was applied across the 12 primary continuous outcomes, yielding a corrected significance threshold of α=0.05/12 = 0.0042; categorical outcomes were not included in this correction. (6) This was a complete-case analysis restricted to patients with complete baseline and follow-up data, following an as-treated principle; P<0.05 (uncorrected) or P<0.0042 (after Bonferroni correction) was considered statistically significant.

## 3 Results

### 3.1 Baseline characteristics

The two groups were generally similar in age, sex, BMI, diabetes duration, comorbidities, smoking history, insulin use, number of concomitant medications, and the main classes of glucose-lowering, lipid-lowering, and antihypertensive medication (**Tables 1**, **2**). During follow-up, the IG showed more frequent initiation or intensification of SGLT2 inhibitors, GLP-1 receptor agonists, and intensified lipid-lowering therapy, together with more pronounced reduction or discontinuation of sulfonylureas and reduction in daily insulin dose; medication-regimen changes were comparatively less frequent in the CG. These medication adjustments were part of the intervention pathway and may represent a mediating factor underlying the observed differences in outcomes.

**Table 1:** Baseline characteristics of the two groups (mean ± SD/[n(%)]).

Variable	IG (n=85)	CG (n=78)	t/χ²	P
Age (years)	61.27 ± 7.76	62.82 ± 6.13	−1.407	0.161
Male sex, n(%)	45 (52.94)	38 (48.72)	0.146	0.702
BMI (kg/m²)	25.91 ± 2.98	25.90 ± 2.73	0.013	0.990
Diabetes duration (years)	8.56 ± 3.88	8.55 ± 3.37	0.028	0.977
Hypertension, n(%)	52 (61.18)	45 (57.69)	0.086	0.770
Dyslipidemia, n(%)	51 (60.00)	43 (55.13)	0.221	0.638
Coronary heart disease, n(%)	13 (15.29)	12 (15.38)	0.000	1.000
Smoking history, n(%)	24 (28.24)	21 (26.92)	0.000	0.991
Insulin use, n(%)	32 (37.65)	36 (46.15)	0.886	0.347
No. of concomitant medications	3.96 ± 1.28	3.81 ± 1.16	0.818	0.414

Data are presented as mean ± SD or n (%). Baseline characteristics were balanced between the two groups; all covariate standardized mean differences (SMDs) were <0.1 after propensity score matching.

**Table 2:** Baseline and 6-month medication regimens.

Medication class/regimen	IG baseline (n=85)	CG baseline (n=78)	IG 6 months (n=85)	CG 6 months (n=78)
Metformin, n(%)	72 (84.71)	65 (83.33)	73 (85.88)	65 (83.33)
Sulfonylureas, n(%)	24 (28.24)	23 (29.49)	17 (20.00)	22 (28.21)
α-glucosidase inhibitors, n(%)	19 (22.35)	18 (23.08)	18 (21.18)	18 (23.08)
Thiazolidinediones, n(%)	5 (5.88)	5 (6.41)	4 (4.71)	5 (6.41)
DPP-4 inhibitors, n(%)	22 (25.88)	21 (26.92)	21 (24.71)	21 (26.92)
SGLT2 inhibitors, n(%)	21 (24.71)	18 (23.08)	30 (35.29)	20 (25.64)
GLP-1 receptor agonists, n(%)	9 (10.59)	8 (10.26)	15 (17.65)	9 (11.54)
Insulin use, n(%)	32 (37.65)	36 (46.15)	31 (36.47)	35 (44.87)
Insulin daily dose (U/d)	31.81 ± 10.42	32.13 ± 10.72	28.10 ± 9.82	31.41 ± 10.25
Statins, n(%)	54 (63.53)	49 (62.82)	62 (72.94)	52 (66.67)
Ezetimibe, n(%)	7 (8.24)	6 (7.69)	12 (14.12)	7 (8.97)
ACEI/ARB, n(%)	43 (50.59)	39 (50.00)	46 (54.12)	40 (51.28)
Calcium channel blockers, n(%)	30 (35.29)	28 (35.90)	32 (37.65)	29 (37.18)
Beta-blockers, n(%)	11 (12.94)	10 (12.82)	12 (14.12)	10 (12.82)
Diuretics, n(%)	10 (11.76)	9 (11.54)	11 (12.94)	10 (12.82)
New/intensified SGLT2 inhibitors during follow-up, n(%)	12 (14.12)	3 (3.85)	–	–
New/intensified GLP-1 receptor agonists during follow-up, n(%)	8 (9.41)	2 (2.56)	–	–
Sulfonylurea dose reduction/discontinuation during follow-up, n(%)	9 (10.59)	3 (3.85)	–	–
Insulin dose reduction ≥10%, n/users(%)	14/32 (43.75)	7/36 (19.44)	–	–
Intensified lipid-lowering therapy, n(%)	14 (16.47)	5 (6.41)	–	–
Intensified antihypertensive therapy, n(%)	11 (12.94)	6 (7.69)	–	–

### 3.2 Glycemic control

**Figure 2:**
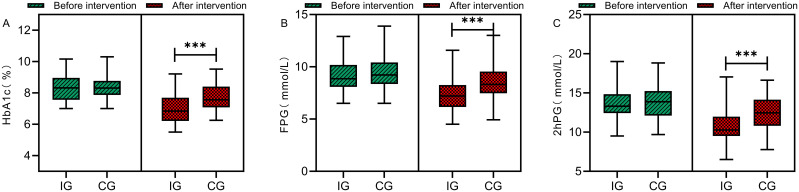
Glycemic indices before and after intervention. After 6 months, HbA1c (panel **A**), FPG (panel **B**), and 2hPG (panel **C**) were all significantly lower in the IG than in the CG (P<0.001). HbA1c, glycated hemoglobin; FPG, fasting plasma glucose; 2hPG, 2-hour postprandial glucose. ***P<0.001.

HbA1c, FPG, and 2hPG did not differ significantly between groups at baseline (all P>0.05). After 6 months of intervention, all three glycemic indices were significantly lower in the IG than in the CG (all P<0.001) (**Table 3**, **Figure 2**).

**Table 3:** Glycemic indices before and after intervention (mean ± SD).

Index	Timepoint	IG (n=85)	CG (n=78)	ANCOVA difference (95% CI)	t	P
HbA1c (%)	Baseline	8.31 ± 0.84	8.29 ± 0.69	—	0.162	0.872
	Post-intervention	6.97 ± 0.90	7.67 ± 0.75	—	−5.394	<0.001
	Post-intervention (ANCOVA-adjusted)	6.96	7.68	−0.72 (−0.847, −0.596)	—	<0.001
FPG (mmol/L)	Baseline	9.15 ± 1.60	9.36 ± 1.53	—	−0.875	0.383
	Post-intervention	7.31 ± 1.68	8.48 ± 1.62	—	−4.530	<0.001
	Post-intervention (ANCOVA-adjusted)	7.41	8.37	−0.96 (−1.120, −0.796)	—	<0.001
2hPG (mmol/L)	Baseline	13.49 ± 2.12	13.72 ± 1.96	—	−0.716	0.475
	Post-intervention	10.71 ± 2.35	12.52 ± 2.06	—	−5.228	<0.001
	Post-intervention (ANCOVA-adjusted)	10.82	12.40	−1.59 (−1.833, −1.339)	—	<0.001

HbA1c, glycated hemoglobin; FPG, fasting plasma glucose; 2hPG, 2-hour postprandial glucose. ANCOVA models used the baseline value of each index, age, and diabetes duration as covariates. Bonferroni-corrected α=0.0042 (0.05/12); all indices remained significant after correction.

### 3.3 Lipid and blood pressure profiles

TC, TG, LDL-C, HDL-C, SBP, and DBP did not differ significantly between groups at baseline (all P>0.05). After 6 months, TC, TG, LDL-C, SBP, and DBP were all significantly lower in the IG, while HDL-C was significantly higher (all P<0.05), suggesting that the multimodal remote medication intervention was associated with broad improvement in cardiometabolic risk factors (**Table 4**).

**Table 4:** Lipid and blood pressure profile before and after intervention (mean ± SD).

Index	Timepoint	IG (n=85)	CG (n=78)	ANCOVA difference (95% CI)	t	P
TC (mmol/L)	Baseline	5.39 ± 0.79	5.33 ± 0.89	—	0.471	0.638
	Post-intervention	4.71 ± 0.85	5.10 ± 0.96	—	−2.794	0.006
	Post-intervention (ANCOVA-adjusted)	4.68	5.13	−0.46 (−0.549, −0.368)	—	<0.001
TG (mmol/L)	Baseline	2.29 ± 0.78	2.34 ± 0.76	—	−0.384	0.702
	Post-intervention	1.69 ± 0.82	2.16 ± 0.83	—	−3.669	<0.001
	Post-intervention (ANCOVA-adjusted)	1.71	2.13	−0.43 (−0.528, −0.326)	—	<0.001
LDL-C (mmol/L)	Baseline	3.35 ± 0.78	3.40 ± 0.67	—	−0.414	0.680
	Post-intervention	2.66 ± 0.79	3.09 ± 0.70	—	−3.746	<0.001
	Post-intervention (ANCOVA-adjusted)	2.68	3.07	−0.39 (−0.483, −0.305)	—	<0.001
HDL-C (mmol/L)	Baseline	1.15 ± 0.22	1.11 ± 0.20	—	1.109	0.269
	Post-intervention	1.27 ± 0.24	1.15 ± 0.21	—	3.156	0.002
	Post-intervention (ANCOVA-adjusted)	1.25	1.17	+0.08 (0.047, 0.106)	—	<0.001
SBP (mmHg)	Baseline	144.59 ± 12.64	141.90 ± 13.01	—	1.338	0.183
	Post-intervention	129.49 ± 13.89	136.05 ± 14.19	—	−2.980	0.003
	Post-intervention (ANCOVA-adjusted)	128.19	137.47	−9.28 (−10.929, −7.638)	—	<0.001
DBP (mmHg)	Baseline	86.15 ± 7.90	88.69 ± 9.14	—	−1.902	0.059
	Post-intervention	77.61 ± 9.03	84.88 ± 9.47	—	−5.017	<0.001
	Post-intervention (ANCOVA-adjusted)	78.83	83.55	−4.72 (−5.807, −3.632)	—	<0.001

TC, total cholesterol; TG, triglycerides; LDL-C, low-density lipoprotein cholesterol; HDL-C, high-density lipoprotein cholesterol; SBP, systolic blood pressure; DBP, diastolic blood pressure. ANCOVA models used the baseline value of each index, age, and diabetes duration as covariates. Bonferroni-corrected α=0.0042 (0.05/12); all indices remained significant after correction.

### 3.4 Insulin resistance and β-cell function

**Figure 3:**
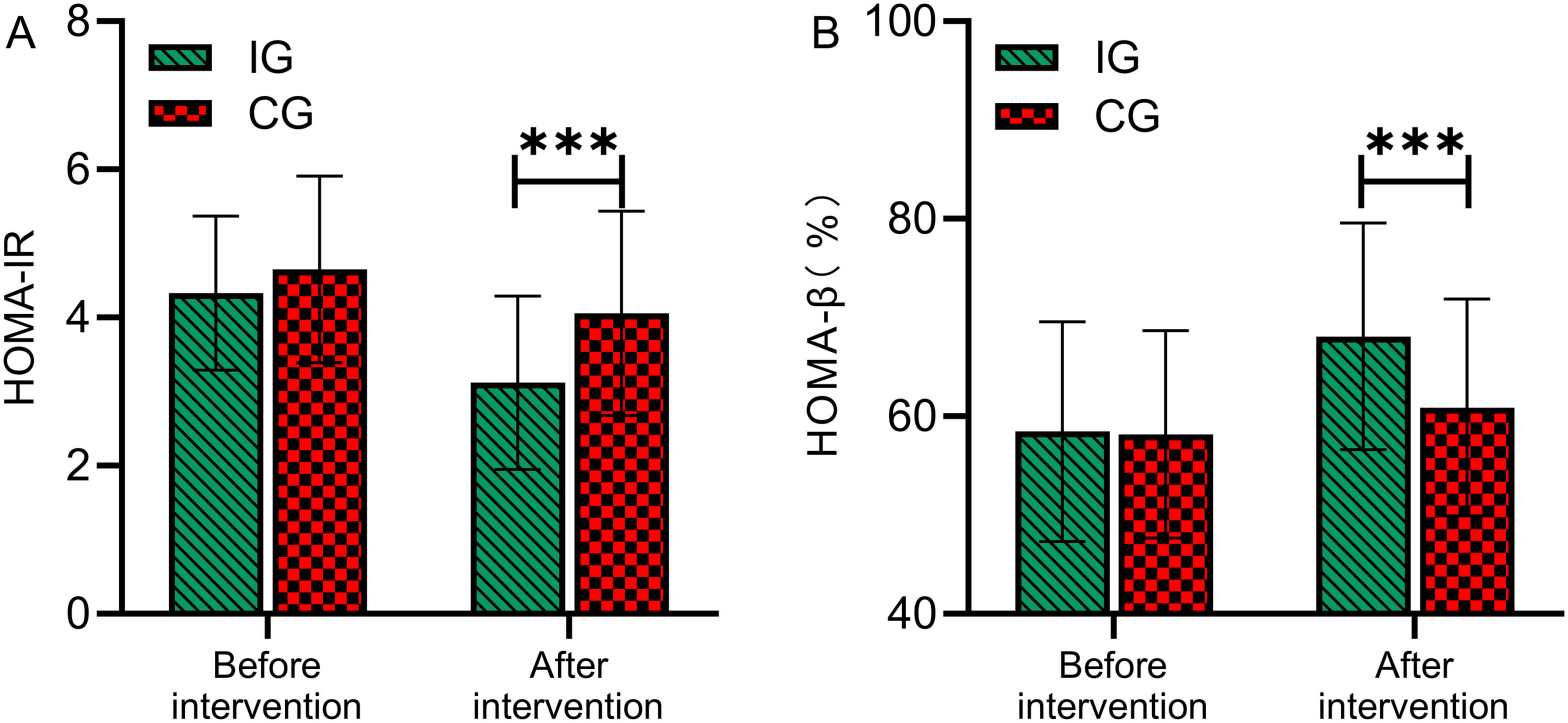
Insulin resistance and β-cell function before and after intervention. After intervention, HOMA-IR (panel **A**) was significantly lower and HOMA-β (panel **B**) significantly higher in the IG than in the CG (P<0.001). ***P<0.001.

HOMA-IR and HOMA-β did not differ significantly between groups at baseline (both P>0.05). At 6 months, HOMA-IR was lower and estimated HOMA-β was higher in the IG than in the CG (both P<0.001) (**Table 5**, **Figure 3**). These between-group differences in surrogate indices do not establish a causal mechanism of improved insulin resistance or recovery of β-cell function.

**Table 5:** Insulin resistance and β-cell function indices before and after intervention (mean ± SD).

Index	Timepoint	IG (n=85)	CG (n=78)	ANCOVA difference (95% CI)	t	P
HOMA-IR	Baseline	4.33 ± 1.04	4.65 ± 1.26	—	−1.741	0.084
	Post-intervention	3.12 ± 1.17	4.06 ± 1.38	—	−4.716	<0.001
	Post-intervention (ANCOVA-adjusted)	3.27	3.90	−0.63 (−0.792, −0.462)	—	<0.001
HOMA-β (%)	Baseline	58.46 ± 11.13	58.17 ± 10.50	—	0.174	0.862
	Post-intervention	68.10 ± 11.46	60.90 ± 10.96	—	4.091	<0.001
	Post-intervention (ANCOVA-adjusted)	67.96	61.05	+6.92 (5.594, 8.238)	—	<0.001

HOMA-IR, homeostatic model assessment of insulin resistance; HOMA-β, homeostatic model assessment of β-cell function. ANCOVA models used the baseline value of each index, age, and diabetes duration as covariates. Bonferroni-corrected α=0.0042 (0.05/12); all indices remained significant after correction.

### 3.5 PSM cohort and IPTW results

ANCOVA results for the 59 matched pairs after PSM (**Table 6**) showed that all 12 outcomes remained significant after Bonferroni correction, fully consistent with the ANCOVA findings in the full cohort. In the IPTW-weighted analysis, 10 of 12 outcomes remained significant after Bonferroni correction (TC and SBP approached significance, P = 0.005 and P = 0.006, respectively), with directions fully consistent with the primary analysis; results from the three analytic approaches were highly concordant.

**Table 6:** PSM-cohort (59 pairs) ANCOVA results compared with the full cohort.

Outcome	Full-cohort ANCOVA difference	PSM-cohort ANCOVA difference	IPTW-weighted difference
HbA1c (%)	−0.72*	−0.74*	−0.72*
FPG (mmol/L)	−0.96*	−0.98*	−1.26*
2hPG (mmol/L)	−1.59*	−1.54*	−1.84*
TC (mmol/L)	−0.46*	−0.47*	−0.40 ns
TG (mmol/L)	−0.43*	−0.51*	−0.42*
LDL-C (mmol/L)	−0.39*	−0.38*	−0.46*
HDL-C (mmol/L)	+0.08*	+0.06*	+0.12*
SBP (mmHg)	−9.28*	−9.31*	−6.08 ns
DBP (mmHg)	−4.72*	−4.58*	−6.64*
HOMA-IR	−0.63*	−0.57*	−0.97*
HOMA-β (%)	+6.92*	+7.06*	+6.87*
MMAS-8 (points)	+0.84*	+0.85*	+1.15*

*, significant after Bonferroni correction (P<0.0042); ns, not significant after Bonferroni correction but P<0.05; PSM, propensity score matching; IPTW, inverse probability of treatment weighting.

### 3.6 Medication adherence and intervention engagement

MMAS-8 scores were comparable between groups at baseline (P>0.05). After intervention, MMAS-8 scores were significantly higher in the IG (P<0.001). The proportion of patients with high adherence rose to 44.71% in the IG versus 20.51% in the CG; the proportion with low adherence in the IG (16.47%) was significantly lower than in the CG (61.54%). The overall distribution of post-intervention adherence categories differed significantly between groups (χ²=35.053, P<0.001) (**Table 7**). Further analysis showed that 71 IG patients (83.53%) achieved a high or moderate level of participation (MMAS-8 ≥6), with only 16.47% classified as low participation; 67 patients (78.82%) showed an improvement in MMAS-8 of ≥1 point from baseline, with a mean improvement of 1.45 ± 0.49 points.

**Table 7:** Medication adherence scores and categories.

Variable	IG (n=85)	CG (n=78)	t/χ²	P
MMAS-8 baseline (mean ± SD, points)	5.43 ± 0.97	5.15 ± 1.19	1.667	0.097
MMAS-8 post-intervention (mean ± SD, points)	6.88 ± 0.89	5.80 ± 1.21	6.530	<0.001
Post-intervention (ANCOVA-adjusted)	6.77	5.93	+0.84 (0.675, 1.010)	<0.001
High adherence (>7 points), n(%)	38 (44.71)	16 (20.51)	35.053	<0.001
Moderate adherence (6–7 points), n(%)	33 (38.82)	14 (17.95)	—	—
Low adherence (<6 points), n(%)	14 (16.47)	48 (61.54)	—	—

MMAS-8, Morisky Medication Adherence Scale–8 items. Post-intervention adherence category distribution was compared by overall chi-square test. ANCOVA used the baseline value of each index, age, and diabetes duration as covariates. Bonferroni-corrected α=0.0042 (0.05/12); all indices remained significant after correction.

### 3.7 Individualized treatment target attainment and drug-related adverse events

HbA1c attainment, LDL-C attainment, and composite triple-target attainment rates were all significantly higher in the IG than in the CG (all P<0.01). Blood pressure attainment did not reach statistical significance (P = 0.246), although the IG numerically outperformed the CG (56.47% vs. 46.15%). During follow-up, the incidence of mild hypoglycemia, severe hypoglycemia, skin reactions, and hypoglycemia-related emergency visits or hospitalizations was lower in the IG than in the CG, with the difference in hypoglycemia-related emergency visits or hospitalizations reaching statistical significance (P = 0.027). The overall incidence of drug-related adverse events was significantly lower in the IG (16.47%) than in the CG (32.05%) (χ²=4.603, P = 0.032) (**Table 8**). Given the small number of events, the absence of multiplicity correction, and the retrospective source of the data, these safety findings should be regarded as exploratory.

**Table 8:** Individualized treatment target attainment and drug-related adverse events [n(%)].

Target/event	IG (n=85)	CG (n=78)	χ²/Fisher	P
HbA1c <7.0%	61 (71.76)	26 (33.33)	22.621	<0.001
Blood pressure <130/80 mmHg	48 (56.47)	36 (46.15)	1.345	0.246
LDL-C attainment	53 (62.35)	27 (34.62)	11.437	<0.001
Composite triple-target attainment	31 (36.47)	11 (14.10)	9.502	0.002
Hypoglycemia	4 (4.71)	10 (12.82)	Fisher	0.092
Severe hypoglycemia	0 (0.00)	2 (2.56)	Fisher	0.227
Gastrointestinal adverse reactions	8 (9.41)	4 (5.13)	Fisher	0.375
Skin reactions	2 (2.35)	6 (7.69)	Fisher	0.154
Hypoglycemia-related ED visit/hospitalization	2 (2.35)	9 (11.54)	Fisher	0.027
Overall drug-related adverse event rate	14 (16.47)	25 (32.05)	4.603	0.032

Composite triple-target attainment required simultaneous achievement of the HbA1c, blood pressure, and LDL-C targets. LDL-C target: <1.8 mmol/L for patients with established ASCVD or high cardiovascular risk; <2.6 mmol/L for others.

### 3.8 Subgroup analyses

Pre-specified exploratory subgroup analyses are presented in **Tables 9**–**11**. In the age-stratified analysis, after ANCOVA adjustment, HbA1c and MMAS-8 were more favorable in the IG than in the CG within both the <65-year and ≥65-year strata. When stratified by insulin use, the direction of the between-group differences in HbA1c, MMAS-8, and HOMA-IR was generally consistent regardless of insulin use. When stratified by baseline adherence, lower follow-up HbA1c and higher MMAS-8 in the IG than in the CG were observed within both the low-adherence and moderate-to-high-adherence strata. Because no formal interaction tests were performed, and because baseline imbalances were present in some subgroups, these results indicate only the direction of the within-subgroup association and cannot be used to conclude that elderly patients, insulin users, or patients with low adherence derive greater benefit.

**Table 9:** Exploratory subgroup comparison of primary outcomes by age stratum (ANCOVA-adjusted).

Index	<65 yr IG (n=52)	<65 yr CG (n=48)	P	≥65 yr IG (n=33)	≥65 yr CG (n=30)	P
HbA1c baseline (%)	8.45 ± 0.81	8.11 ± 0.70	0.026*	8.08 ± 0.85	8.58 ± 0.56	0.008*
HbA1c ANCOVA-adjusted mean (%)	7.01	7.66	<0.001†	6.92	7.69	<0.001
ANCOVA-adjusted difference (95% CI)	−0.649 (−0.813, −0.485)	—	<0.001	−0.772 (−0.987, −0.558)	—	<0.001
MMAS-8 ANCOVA-adjusted mean (points)	6.67	5.69	<0.001	6.92	6.30	<0.001†

ANCOVA used baseline HbA1c as the covariate for the adjusted comparison of post-intervention values between the IG and CG within each subgroup; adjusted means and ANCOVA between-group differences (95% CI) are reported. †P value from the ANCOVA F test (HbA1c: F(1,97)=60.23; ≥65-year group F(1,60)=49.80; MMAS-8: F(1,97)=74.37; ≥65-year group F(1,60)=23.37). *Indicates baseline HbA1c imbalance between groups within the stratum (P<0.05), which was addressed by ANCOVA covariate adjustment.

**Table 10:** Exploratory subgroup comparison of primary outcomes by insulin use (ANCOVA-adjusted).

Index	Insulin IG (n=32)	Insulin CG (n=36)	P	No-insulin IG (n=53)	No-insulin CG (n=42)	P
HbA1c ANCOVA-adjusted mean (%)	7.03	7.72	<0.001†	6.92	7.65	<0.001
MMAS-8 ANCOVA-adjusted mean (points)	6.78	6.11	<0.001†	6.77	5.77	<0.001
HOMA-IR ANCOVA-adjusted mean	3.20	3.81	<0.001†	3.31	3.96	<0.001

ANCOVA used the corresponding baseline value of each outcome (HbA1c, MMAS-8, HOMA-IR) as the covariate. †P value from the ANCOVA F test (HbA1c insulin group F(1,65)=44.60, no-insulin group F(1,92)=79.41; MMAS-8 insulin group F(1,65)=22.63, no-insulin group F(1,92)=93.41; HOMA-IR insulin group F(1,65)=16.57, no-insulin group F(1,92)=41.12); all P<0.001.

**Table 11:** Exploratory subgroup comparison of primary outcomes by baseline medication adherence (ANCOVA-adjusted).

Index	Low adherence IG (n=63)	Low adherence CG (n=61)	P	Moderate-high adherence IG (n=22)	Moderate-high adherence CG (n=17)	P
HbA1c ANCOVA-adjusted mean (%)	6.95	7.70	<0.001	7.00	7.61	<0.001†
MMAS-8 ANCOVA-adjusted mean (points)	6.41	5.50	<0.001	7.92	7.31	<0.001

ANCOVA used the corresponding baseline value of each outcome as the covariate. Low adherence defined as baseline MMAS-8 <6; moderate-to-high adherence defined as baseline MMAS-8 ≥6. †P value from the ANCOVA F test (HbA1c low-adherence group F(1,121)=94.12, moderate-to-high group F(1,36)=28.09; MMAS-8 low-adherence group F(1,121)=71.21, moderate-to-high group F(1,36)=39.39).

## 4 Discussion

### 4.1 Association with glycemic control

In this study, HbA1c in the IG declined from 8.31% to 6.97% over 6 months, compared with a decline from 8.29% to 7.67% in the CG, with an ANCOVA-adjusted between-group difference of −0.72 percentage points. This finding is directionally consistent with systematic reviews, randomized trials, and collaborative-care studies of pharmacist-led diabetes management (9, 18–21). However, because this was a non-randomized retrospective study in which group allocation was accompanied by differences in the intensity, degree of structuring, and continuity of pharmacist contact, the observed difference cannot be attributed solely to the ‘remote’ format or to the multimodal combination itself. Application-based reminders, video follow-up, real-time alerts, and individualized review may plausibly reduce missed doses and facilitate timely regimen adjustment; however, this study did not measure the dose or frequency of exposure to each component, nor did it examine mediating pathways, so these explanations remain reasonable hypotheses that cannot be directly confirmed by the present data.

### 4.2 Mechanistic pathways: insulin resistance and β-cell function

HOMA-IR was lower and estimated HOMA-β was higher in the IG at follow-up. Both indices are derived from fasting glucose and fasting insulin and are indirect surrogate measures that may be influenced by medication regimen, recent glycemic status, and assay variability in insulin measurement. Accordingly, these findings can only be described as an association with a lower estimated insulin-resistance index and a higher estimated β-cell function index, and do not establish ‘recovery’ of β-cell function or a biological mechanism. Diet, physical activity, weight management, improved adherence, and optimization of medication regimens may all have contributed to these changes (22–24); however, this study did not perform mediation analysis and lacked complete data on medication class and dose changes, so any mechanistic interpretation should be regarded as hypothesis-generating.

### 4.3 Effects on medication adherence

Medication adherence is a key determinant of therapeutic success in T2DM. MMAS-8 scores in the IG rose from 5.43 to 6.88 points, and the proportion of patients with high adherence rose from under 20% to 44.71%, significantly exceeding the 20.51% observed in the CG. These results are consistent with a randomized controlled trial by Poonprapai et al. (25), which found that pharmacist-delivered, mobile application–based family support significantly improved diabetes knowledge, self-management behavior, and medication adherence in older adults with diabetes. Christy et al. (26) similarly showed that pharmacist-led digital health interventions reduced missed doses and improved medication regularity. In this study, the improvement in adherence appeared to operate at several levels: behaviorally, application and instant-messaging reminders may help reinforce regular medication-taking habits; psychologically, pharmacist encouragement during video consultations may strengthen treatment confidence and alleviate medication-related anxiety; educationally, individualized counseling improved patients’ understanding of drug mechanisms and adverse effects, which may reduce self-discontinuation (27); and at the regimen level, simplification of complex regimens may reduce the burden of polypharmacy. Improved adherence is plausibly linked to more stable drug exposure and more consistent therapeutic effect.

### 4.4 Effects on individualized treatment target attainment

Contemporary diabetes management has shifted from a focus on glycemic control alone toward a comprehensive strategy that simultaneously targets HbA1c, blood pressure, and lipid goals (28). HbA1c attainment reached 71.76%, LDL-C attainment 62.35%, and composite triple-target attainment 36.47% in the IG—all significantly higher than in the CG. The absence of a statistically significant between-group difference in blood pressure attainment may reflect the fact that this intervention was centered primarily on glucose-lowering medication management, with comparatively less emphasis on antihypertensive regimen adjustment; future multimodal interventions should more explicitly incorporate blood pressure management into the intervention pathway. These findings are broadly consistent with the meta-analysis by Alabkal et al. (10), which confirmed the effectiveness of pharmacist interventions in reducing HbA1c and SBP while also noting ongoing challenges in achieving comprehensive multifactorial risk-factor control. Key elements of individualized target attainment include setting patient-specific HbA1c goals based on age, life expectancy, and comorbidities (4); selecting glucose-lowering agents according to cardiovascular risk stratification, with priority given to agents with demonstrated cardio- and renoprotective properties (SGLT-2 inhibitors or GLP-1 receptor agonists); identifying and managing drug–drug interactions in patients receiving complex polypharmacy; and incorporating patient preference and economic considerations into shared decision-making.

### 4.5 Safety profile

The proportion of patients with documented drug-related adverse events was lower in the IG, and hypoglycemia-related emergency visits or hospitalizations were also less frequent. This direction is consistent with studies of structured pharmacist-led hypoglycemia risk management (29). However, the total number of events in this study was small, the data relied on retrospective medical records and reporting systems, and no standardized causality assessment of adverse drug reactions was performed, so under-reporting and detection bias cannot be excluded. Greater intensity of pharmacist contact could, in theory, either reduce the occurrence of events or increase the likelihood of their detection, and this study cannot distinguish between these possibilities. Accordingly, the safety findings should be regarded as exploratory associations and cannot be used to conclude that the intervention reduced drug-related adverse events or to confirm a safety advantage.

### 4.6 Exploratory subgroup analyses

Within the age-stratified analysis, more favorable HbA1c and MMAS-8 values in the IG relative to the CG were observed in both the <65-year and ≥65-year strata. However, the two age strata showed baseline HbA1c imbalance in opposite directions, and no formal test of the age-by-group interaction was performed; therefore, these results cannot support a conclusion that the intervention was ‘non-inferior’ or more beneficial in older patients than in younger patients. These findings suggest only that the primary association was not confined to a single age stratum and require confirmation in prospective, stratified studies.

Within the insulin-use and non-insulin-use strata, the direction of the within-subgroup comparisons for HbA1c, MMAS-8, and HOMA-IR in the IG relative to the CG was generally consistent. Although some numerical differences appeared larger among non-insulin users, the absence of a formal interaction test precludes any conclusion that non-insulin users or insulin users derived a greater association-level benefit. Treatment complexity, baseline medication regimen, and self-management burden may influence these results, but the present data are insufficient to test these explanations.

Within both the low-adherence and moderate-to-high-adherence strata at baseline, lower follow-up HbA1c and higher MMAS-8 were observed in the IG. These results may generate hypotheses for future adherence-stratified studies but do not demonstrate that patients with low baseline adherence constitute the subgroup deriving the greatest benefit. Future studies should pre-specify interaction tests and use objective adherence and digital-platform engagement metrics to determine whether true effect heterogeneity exists.

### 4.7 Clinical implications and limitations

This study describes, across multiple dimensions—glycemic control, lipid profile, blood pressure, HOMA-based estimates, medication adherence, and composite target attainment—the association between a multimodal remote pharmaceutical service and clinical outcomes. The directional consistency of the PSM and IPTW results supports the robustness of the primary findings after adjustment for measured confounders, but cannot eliminate unmeasured confounding. The 92.02% rate of physician adoption of pharmacist recommendations may reflect the feasibility of the physician–pharmacist collaborative pathway but does not, on its own, establish clinical effectiveness. Recent literature has emphasized the value of pharmacists in medication optimization, adherence support, pharmacovigilance, and interprofessional collaboration (20, 28, 29); taken together with the present findings, a more cautious clinical interpretation is that structured, continuous, and integrated pharmacist support may be associated with more favorable T2DM management outcomes, although whether this advantage derives from the remote technology, the multimodal combination, or the greater intensity of contact cannot be distinguished.

This study has the following limitations. First, as a single-center retrospective study, the sample size was relatively limited; although PSM (which further reduced the sample size and statistical power relative to the full-cohort analysis) and IPTW were used to control for observable confounders, residual confounding cannot be fully excluded, and the generalizability of the findings is correspondingly limited. Second, the control and intervention groups differed in the intensity, degree of structuring, and continuity of service, so the independent contributions of the remote delivery channel, the multimodal combination, and contact intensity cannot be fully disentangled. Third, although the reference protocol specified contact frequency, reminder rules, training, and quality-control procedures, retrospective records may still have missed patient-initiated inquiries, brief instant-messaging contacts, and interactions that were not formally documented, and fidelity of implementation was not assessed by an independent third party. Fourth, the supplementary analysis of baseline and follow-up medication regimens improves the interpretability of the findings, but medication adjustment is itself a time-varying component of the intervention and a potential mediating variable; this study did not use marginal structural models or formal mediation analysis and therefore cannot quantify the independent contribution of individual medication adjustments to the outcomes. Fifth, HOMA-IR and HOMA-β are both indirect surrogate indices and cannot establish recovery of insulin sensitivity or β-cell function. Sixth, the number of drug-related adverse events was small and relied on retrospective documentation, which may be subject to under-reporting and detection bias. Seventh, the subgroup analyses did not include formal interaction tests. Eighth, the inclusion requirement of proficient smartphone use may have introduced selection bias related to digital accessibility. Ninth, the 6-month follow-up cannot evaluate long-term hard endpoints, and no cost-effectiveness analysis was performed. Future research should include multicenter, prospective, randomized or factorial studies that pre-specify component-level exposure, documentation of medication adjustments, and independent quality audits.

## 5 Conclusion

In this single-center retrospective observational study, a clinical pharmacist-led multimodal remote medication intervention was associated with more favorable glycemic and cardiometabolic indices, lower HOMA-IR, higher estimated HOMA-β, higher medication adherence, and higher attainment of individualized treatment targets; the overall direction of the PSM and IPTW analyses was consistent with the primary analysis. Because of the non-randomized group allocation, the unequal intensity of service contact between groups, incomplete documentation of medication-regimen changes, the limitations of HOMA-based surrogate indices, the retrospective ascertainment of drug-related adverse events, and the absence of subgroup interaction testing, this study cannot establish causality, recovery of β-cell function, the independent effect of any single intervention component, a safety advantage, or greater benefit in any specific subgroup. This service model may warrant further structured evaluation in institutions with established digital infrastructure and multidisciplinary collaboration, and multicenter prospective studies are needed to confirm its effectiveness, feasibility, and cost-effectiveness.

## Data Availability

The original contributions presented in the study are included in the article/supplementary material, further inquiries can be directed to the corresponding author/s.
